# Long-Term High-Altitude Exposure, Accelerated Aging, and Multidimensional Aging-Related Changes

**DOI:** 10.1001/jamanetworkopen.2025.9960

**Published:** 2025-05-13

**Authors:** Yuwei Wu, Yuming Jin, Linghui Deng, Yinlong Wang, Yurui Wang, Junhan Chen, Ruohan Gao, Shichao Wei, Guohua Ni, Xianghong Zhou, Zilong Zhang, Bin Zeng, Chuzhong Wei, Weichao Huang, Shi Qiu, Birong Dong

**Affiliations:** 1 Department of Urology, Institute of Urology and National Clinical Research Center for Geriatrics, West China Hospital of Sichuan University, Chengdu, Sichuan Province, China; 2 Department of Geriatrics, National Clinical Research Center for Geriatrics, West China Hospital, Sichuan University, Chengdu, China; 3 West China Hospital of Sichuan University, Chengdu, Sichuan Province, People’s Republic of China; 4 High Altitude Medicine Key Laboratory of Sichuan Province, West China Hospital, Sichuan University, Chengdu, Sichuan, China; 5 West China Biomedical Big Data Center, West China Hospital, Sichuan University, Chengdu, China

## Abstract

**Importance:**

Exposure to high altitudes elicits multiple adaptive mechanisms that intricately impact the entire body, causing deleterious health outcomes. However, high-altitude exposure effects on accelerated aging and aging-related changes remain uncertain.

**Objective:**

To comprehensively assess the associations of high-altitude exposure with overall aging and related changes and to provide insights into the treatment and prevention of aging-associated deficits in populations living in high-altitude areas.

**Design, Setting, and Participants:**

This population-based cross-sectional study used data from 2 prospective studies in Western China: West China Natural Population Cohort (WCNPCS) and West China Health and Aging Trend (WCHAT). The WCNPCS cohort was constructed from May 2019 to June 2021. Data were collected from participants aged 18 years and older in 4 populous regions (Mianzhu, Longquan, Pidu, and Ganzi) in Sichuan Province. The WCHAT was initiated in 2018 and recruited participants aged 50 years and older from various regions (Sichuan, Yunnan, Guizhou, and Xinjiang). Participants were selected via sequential cluster sampling from the permanent residents of the participating community. Data for the present study were analyzed between March and October 2024.

**Exposure:**

The participants’ altitudes were determined using the global Shuttle Radar Topography Mission 4 data based on residential addresses. High-altitude areas refer to regions with altitudes of greater than or equal to 1500 m (4921 feet) above the mean sea level.

**Main Outcomes and Measures:**

Biological aging (BA) and aging acceleration (AA) were measured through the Klemera–Doubal Biological Age (KDM-BA) and PhenoAge methods. Multidimensional aging-related metrics were based on questionnaire, measurement, and self-report.

**Results:**

A total of 9846 participants from the WCNPCS cohort (mean [SD] age, 55.73 [11.06] years; 6730 women [68.35%]) and 3593 participants from the WCHAT cohort (mean [SD] age, 62.27 [8.40] years; 2253 women [62.71%]) were included. The participants living at high altitudes presented increased KDM-BA acceleration by 0.85 years for the WCNPCS cohort and 0.71 years for the WCHAT cohort. The PhenoAge results were similar, with even larger effect sizes (WCNPCS, β, 2.08 years; 95% CI, 1.77-2.39 years; WCHAT, β, 2.23 years; 95% CI, 1.91-2.54 years). The association between high-altitude exposure and biologically accelerated aging was particularly pronounced among smokers. Associations between high-altitude exposure and various multidimensional aging-related metrics were also observed.

**Conclusions and Relevance:**

These findings suggest that extended periods at high altitudes may hasten BA and contribute to the onset of aging-related illnesses. Implementing public health interventions for individuals residing in high-altitude regions may aid in alleviating the disease burden within these communities.

## Key Points

**Question:**

Is long-term high-altitude exposure associated with aging and aging-related changes?

**Findings:**

This cross-sectional study of 2 cohorts in Western China, West China Natural Population Cohort (WCNPCS; n = 9846) and West China Health and Aging Trend (WCHAT; n = 3593), found that extended periods at high altitudes were associated with faster biological aging and may contribute to the onset of aging-related illnesses. According to the Klemera–Doubal Biological Age method, age accelerated by 0.85 years in the WCNPCS cohort and 0.71 years in the WCHAT cohort; according to the PhenoAge method, age accelerated by 2.08 years in the WCNPCS cohort and by 2.23 years in the WCHAT cohort.

**Meaning:**

These findings suggest that implementing public health interventions for individuals residing in high-altitude regions may aid in alleviating the disease burden within these communities and, thus, are imperative.

## Teaser

This cross-sectional study assesses the associations of high-altitude exposure with overall aging and aging-related changes among adults in China.

## Introduction

More than 81.6 million individuals worldwide permanently reside in high-altitude regions (conventionally defined as >1500 m).^1^ In these regions, several activities, including sports, training, tourism, scientific research, work, and military operations, have increased greatly in recent years. Compared with sea-level regions, high-altitude regions have reduced atmospheric oxygen levels, diminished air pressure, and intense ultraviolet radiation.^2^ Exposure to high altitudes elicits various adaptive mechanisms that intricately impact the entire cardiovascular, respiratory, and central nervous systems, leading to deleterious health outcomes. Recently, using a Chinese multiethnic cohort comprising nearly 100 000 participants, Zuo et al^3^ found that high-altitude exposure may lead to bone mineral density reduction among adults, especially the older ones. Burtscher et al^4^ also concluded that high-altitude exposure can impair the cognitive performance of older individuals—a manifestation of brain aging. However, the associations of high-altitude exposure with more aging-related changes, especially the overall aging of humans, remain uncertain.

The aging process, which is multifactorial and inevitable, results in a decline in organ functions and changes in multidimensional aging-related metrics. If it is accelerated, the risks of chronic diseases and death increase. Moreover, accelerated aging and related diseases pose substantial challenges to contemporary medical care, the economy, and society as a whole, thereby emerging as a global public health priority.^5^ Chronological age remains constant and does not accurately reflect the aging status. Hence, biological aging (BA) is a better and modifiable indicator of aging than chronological aging; BA can either lag behind or exceed chronological age. Ideal BA assessments should encompass wide-ranging aging-related changes across multiple systems, including biomarkers, such as telomere length, and algorithms that integrate data from various molecular levels.^6^ Among these, the Klemera–Doubal Biological Age (KDM-BA) method and the PhenoAge algorithm incorporate data from standard clinical parameters and have demonstrated exceptional accuracy in estimating morbidity and mortality.^7^ Therefore, we used the KDM-BA and PhenoAge algorithms to compute participants’ BA based on the West China Natural Population Cohort (WCNPCS) study and the West China Health and Aging Trend (WCHAT) study. Thereafter, we examined the associations of high-altitude exposure with accelerated BA and multidimensional aging-related metrics. This study aimed to comprehensively assess the associations of high-altitude exposure with overall aging and related changes through different dimensions, providing valuable insights into the treatment and prevention of aging-associated deficits in populations living in high-altitude areas.

## Methods

### Study Population

This cross-sectional study used data from 2 cohorts in Western China: WCNPCS and WCHAT. The WCNPCS cohort was constructed from May 2019 to June 2021.^8^ Data were collected from 4 populous regions (Mianzhu, Longquan, Pidu, and Ganzi) in Sichuan Province, Western China. The WCNPCS recruited participants aged 18 years and older, and participants were selected via sequential cluster sampling from the permanent residents of the participating community. Trained medical staff conducted face-to-face interviews, physical examinations, biological sampling, including serum and urine, and special examinations.

The WCHAT was initiated in 2018 and recruited participants aged 50 years and older from various regions (Sichuan, Yunnan, Guizhou, and Xinjiang) in Western China on the basis of preestablished criteria. Annual follow-ups were subsequently conducted through onsite visits (in 2019, 2021, 2022, and 2023) or telephone interviews (in 2020). The onsite follow-ups included questionnaire surveys, physical examinations, and laboratory examinations. Further details on the cohort profile are available in previously published literature.^9^

**Figure 1.**
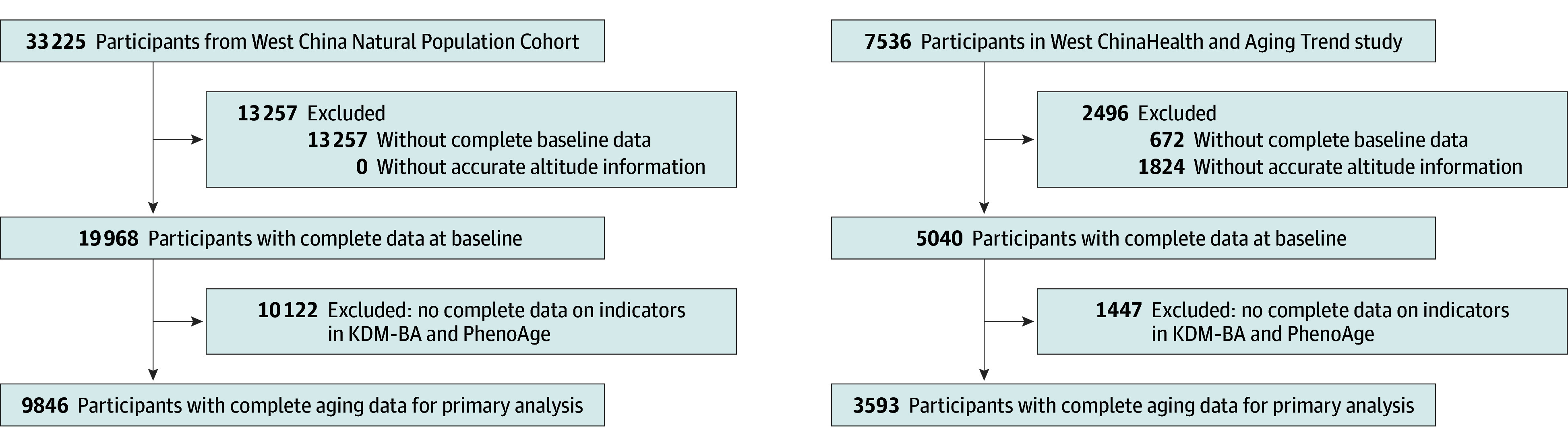
Study Enrollment Flowchart KDM-BA indicates Klemera–Doubal Biological Age.

The WCNPCS initially enrolled 33 225 participants. Those who had no altitude information (0 participants), KDM-BA or PhenoAge data (10 122 participants), and adjusted covariates (13 257 participants) were excluded. In the WCHAT cohort, 7536 participants were enrolled. Those who lacked altitude information (1824 participants), KDM-BA or PhenoAge (1447 participants), and adjusted covariates (672 participants) were also excluded (Figure 1). In addition, the WCHAT cohort provided baseline multidimensional aging-related metrics that were used to further evaluate the associations between high-altitude exposure and aging-related changes. All participants were voluntarily recruited, providing written informed consent before the survey. The study protocols were approved by the ethics committee of West China Hospital, Sichuan University. Both studies were registered with the China Clinical Trial Registration Center. The Strengthening the Reporting of Observational Studies in Epidemiology (STROBE) reporting guidelines for cross-sectional studies were followed.

### Measurements of Altitude

The altitude data were obtained from the Shuttle Radar Topography Mission version 4, covering China at a spatial resolution of 3 arcseconds (approximately 90 m). These data were sourced from the CGIAR Consortium for Spatial Information.^10^ Each participant’s altitude was matched with the Shuttle Radar Topography Mission data according to their residential location.^11^ High-altitude areas refer to regions with altitudes of greater than or equal to 1500 m (4921 feet) above the mean sea level.^3,12^ Therefore, the participants were categorized into the following according to this 1500-m threshold: low-altitude and high-altitude groups.

### Measurements of BA and Aging Acceleration

We used the KDM-BA and PhenoAge algorithms to measure the participants’ BA.^13^ KDM-BA was calculated according to the forced expiratory volume in 1 second, systolic blood pressure, and certain blood chemistry parameters (albumin, alkaline phosphatase, blood urea nitrogen, creatinine, C-reactive protein, glycated hemoglobin, and total cholesterol). Moreover, PhenoAge was derived from 9 blood chemistry parameters (albumin, creatinine, C-reactive protein, glucose, mean cell volume, alkaline phosphatase, red cell distribution width, white blood cell count, and lymphocyte proportion).^14^ Because alkaline phosphatase was absent in WCHAT, only 8 blood biomarkers were used for calculation. Details of the calculations and formulas are provided in the eAppendix in Supplement 1.

The BA values were computed using the R package BioAge.^15,16^ To quantify interindividual variations in BA, we calculated the difference between biological age estimation and chronological age, termed *aging acceleration* (AA).

### Measurements of the Covariates

We incorporated variables such as age, sex, body mass index (BMI), marital status, education level, smoking habits, alcohol consumption, physical activity level, and the presence of comorbidities such as hypertension, diabetes, and chronic obstructive pulmonary disease (COPD). To calculate the BMI, we divided the weight in kilograms by the square of height in meters. We then classified the participants as underweight (BMI <18.5), normal weight (BMI 18.5-23.9), overweight (BMI 24.0-27.9), or having obesity (BMI >27.9).^17^ Sociodemographic characteristics and lifestyle factors were assessed using a comprehensive questionnaire survey. Marital status included married vs unmarried, divorced, or widowed,^18^ and education status included primary school, junior high school, high school, college, or graduate.^19^ We defined smoking as the consumption of more than 100 cigarettes throughout an individual’s lifetime, and drinking as the intake of more than 30 g per week within the past 12 months. The physical activity level was divided into less than 1 time per week or 1 time or more per week. These operational definitions have been corroborated by previous studies.^20^ A history of hypertension, diabetes, or COPD was determined according to self-reported information. Additionally, systolic blood pressure greater than or equal to 140 mm Hg and/or diastolic blood pressure greater than or equal to 90 mm Hg at baseline defined hypertension.^21^

### Assessment of the Multidimensional Aging-Related Metrics

The activity of daily living (ADL) scale comprises 14 items that refer to individuals’ basic ADLs (BADL) and instrumental ADLs (IADL). BADLs encompass the necessary abilities for performing routine physical activities, such as feeding, dressing, bathing, moving from bed to chair, using toilets, and maintaining continence. Conversely, IADLs encompass more intricate tasks related to the capacity for independent living. These activities include household chores, meal preparation, grocery shopping, financial management, and medication administration.^22^ To evaluate the participants’ cognitive status, we used the 10-item Short Portable Mental Status Questionnaire. Scores greater than or equal to 3 indicate the presence of impaired cognitive function. Importantly, the participants’ education level must be considered when interpreting these results.^23^ Depression was measured using the 15-item Geriatric Depression Scale. This scale is the most widely used tool for detecting depression, with scores greater than or equal to 5 indicating depression.^24^ In assessing anxiety status, we used the Generalized Anxiety Disorder–7 scale, which has been validated in a primary care setting through extensive patient sampling,^25^ and scores greater than or equal to 5 indicate anxiety. Weakness is a common geriatric condition that is assessed by objectively measuring the handgrip strength. The maximum grip strength of the dominant hand over 2 trials less than or equal to the 20th percentile within 8 sex-by-BMI categories was used as the criterion. We also measured sarcopenia by using the diagnostic algorithm recommended by the Asian Working Group for Sarcopenia, which comprises 3 components: muscle mass, muscle strength, and physical performance.^26,27^ Appendicular skeletal muscle mass (ASM) indices (defined as ASM in kilograms divided by height in meters squared) less than or equal to 7.0 kg/m^2^ in men and less than or equal to 5.7 kg/m^2^ in women indicated low muscle mass. Low grip strength was defined as a threshold of 28 kg for men and 18 kg for women. For sarcopenia, a gait speed less than or equal to 0.8 m/s defined low physical performance. Gastrointestinal and kidney diseases were identified according to self-reported information.

### Statistical Analysis

Data for the present study were analyzed between March and November 2024. In this study, the continuous variables are reported as the means (SDs), whereas the categorical variables are presented as proportions. We used the χ^2^ test (or Fisher exact test) and a *t* test for analyzing the categorical and continuous variables, respectively. To examine the associations between high-altitude exposure and AA, we used multivariate linear regression models and multivariate logistic regression models. Estimates were provided as the β and odds ratio (OR) separately, with a 95% CI. The independent variable was the presence or absence of high-altitude exposure, whereas the dependent variables were the KDM-BA, PhenoAge, and AA. Moreover, we sequentially adjusted covariates, including a crude model (model 1) and fully adjusted models (model 2, adjusted for age, sex, marital status, education, BMI, smoking status, drinking status, hypertension status, diabetes status, COPD status, and physical activity status). We further conducted subgroup analyses according to potential covariates and determined the interaction *P* values via the Wald test. We also comprehensively analyzed the associations between high-altitude exposure and multidimensional aging-related metrics to further demonstrate the robustness of our research findings. All statistical data were analyzed using statistical software packages R version 4.3.1 (R Project for Statistical Computing) and EmpowerStats version 4.1 (X&Y Solutions, Inc). A 2-sided *P* < .05 was considered statistically significant.

Sensitivity analyses are important in evaluating the robustness of the primary findings. First, we regarded AA as a categorical variable with AA equal 0 and AA equal to the 90th percentile (P90), and AA values were further divided into 2 categories: AA (AA >0 or AA >P90) and managing acceleration (AA ≤0 or AA ≤P90). Second, we compared the association between PhenoAge and modified PhenoAge with and without alkaline phosphatase and the associations of these 2 parameters with mortality risk. Third, propensity score matching was performed, including age, gender, marital status, and alcohol consumption, for the high-altitude and low-altitude groups, and we conducted further analysis. Fourth, we regressed the participants’ computed BA on their chronological age and the residual values from this regression were used to gauge BA acceleration. Fifth, we conducted inverse probability weighting to eliminate effect of excluded participants. Sixth, we performed supplementary adjustments for additional covariates, including ethnicity, occupation, nutritional status, and medical insurance, in WCHAT. Seventh, nonlinear associations were analyzed using restricted cubic spline analyses.

## Results

### Population Characteristics

This study included 9846 participants from the WCNPCS cohort (6730 women [68.35%]; mean [SD] age, 55.73 [11.06] years) and 3593 participants from the WCHAT cohort (2253 women [62.71%]; mean [SD] age, 62.27 [8.40] years) (Table 1). In the WCNPS cohort, 8996 participants lived in low-altitude areas and 850 lived in high-altitude areas. In the WCHAT cohort, 929 lived in low-altitude areas and 2664 lived in high-altitude areas. Age, sex, BMI, marital status, education status, smoking status, alcohol intake status, physical activity status, and hypertension significantly differed between these 2 groups. Both the KDM-BA and PhenoAge revealed that participants living at high altitudes were more likely to be biologically older than their low-altitude counterparts.

**Table 1.** Baseline Characteristics of the Study Participants

Characteristics	Participants, No. (%)							
	West China Natural Population Cohort				West China Health and Aging Trend			
	Total (n = 9846)	Low altitude (n = 8996)	High altitude (n = 850)	*P* value	Total (n = 3593)	Low altitude (n = 929)	High altitude (n = 2664)	*P* value
Age, mean (SD), y	55.73 (11.06)	55.60 (11.22)	57.06 (9.12)	<.001	62.27 (8.40)	63.75 (8.81)	61.76 (8.20)	<.001
Klemera–Doubal Biological Age acceleration, mean (SD), y	−2.51 (5.36)	−2.61 (5.25)	−1.48 (6.32)	<.001	−2.55 (5.88)	−3.24 (5.92)	−2.31 (5.84)	<.001
PhenoAge acceleration, mean (SD), y	−7.16 (4.45)	−7.29 (4.36)	−5.76 (5.09)	<.001	1.01 (4.82)	−0.54 (4.91)	1.55 (4.67)	<.001
Sex								
Male	3116 (31.65)	2857 (31.76)	259 (30.47)	.44	1340 (37.29)	326 (35.09)	1014 (38.06)	.11
Female	6730 (68.35)	6139 (68.24)	591 (69.53)		2253 (62.71)	603 (64.91)	1650 (61.94)	
Marital status								
Unmarried	4110 (41.74)	3967 (44.10)	143 (16.82)	<.001	570 (15.86)	122 (13.13)	448 (16.82)	.01
Married	5736 (58.26)	5029 (55.90)	707 (83.18)		3023 (84.14)	807 (86.87)	2216 (83.18)	
Education								
Primary school	6235 (63.33)	5865 (65.20)	370 (43.53)	<.001	2302 (64.07)	536 (57.70)	1766 (66.29)	<.001
Junior school	1665 (16.91)	1364 (15.16)	301 (35.41)		808 (22.49)	279 (30.03)	529 (19.86)	
High school	956 (9.71)	850 (9.45)	106 (12.47)		345 (9.60)	93 (10.01)	252 (9.46)	
College	598 (6.07)	526 (5.85)	72 (8.47)		137 (3.81)	21 (2.26)	116 (4.35)	
Graduate	392 (3.98)	391 (4.35)	1 (0.12)		1 (0.03)	0 (0.00)	1 (0.04)	
Body mass index^a^								
<18.5	194 (1.97)	185 (2.06)	9 (1.06)	<.001	77 (2.14)	15 (1.61)	62 (2.33)	<.001
≥18.5 to <24	4328 (43.96)	4004 (44.51)	324 (38.12)		1319 (36.71)	381 (41.01)	938 (35.21)	
≥24 to <28	3985 (40.47)	3627 (40.32)	358 (42.12)		1429 (39.77)	389 (41.87)	1040 (39.04)	
≥28	1339 (13.60)	1180 (13.12)	159 (18.71)		768 (21.37)	144 (15.50)	624 (23.42)	
Smoking								
No	7963 (80.88)	7263 (80.74)	700 (82.35)	.25	2947 (82.02)	734 (79.01)	2213 (83.07)	.01
Yes	1883 (19.12)	1733 (19.26)	150 (17.65)		646 (17.98)	195 (20.99)	451 (16.93)	
Drinking								
No	6937 (70.46)	6695 (74.42)	242 (28.47)	<.001	2698 (75.09)	706 (76.00)	1992 (74.77)	.46
Yes	2909 (29.54)	2301 (25.58)	608 (71.53)		895 (24.91)	223 (24.00)	672 (25.23)	
Hypertension								
No	8062 (81.88)	7421 (82.49)	641 (75.41)	<.001	2718 (75.65)	702 (75.57)	2016 (75.68)	.95
Yes	1784 (18.12)	1575 (17.51)	209 (24.59)		875 (24.35)	227 (24.43)	648 (24.32)	
Diabetes								
No	9258 (94.03)	8463 (94.08)	795 (93.53)	.52	3340 (92.96)	842 (90.64)	2498 (93.77)	.001
Yes	588 (5.97)	533 (5.92)	55 (6.47)		253 (7.04)	87 (9.36)	166 (6.23)	
Chronic obstructive pulmonary disease								
No	9775 (99.28)	8925 (99.21)	850 (100.00)	.009	3553 (98.89)	914 (98.39)	2639 (99.06)	.09
Yes	71 (0.72)	71 (0.79)	0 (0.00)		40 (1.11)	15 (1.61)	25 (0.94)	
Physical activity								
<1 Time/wk	1108 (11.25)	1091 (12.13)	17 (2.00)	<.001	3226 (89.79)	819 (88.16)	2407 (90.35)	.06
≥1 Time/wk	8738 (88.75)	7905 (87.87)	833 (98.00)		367 (10.21)	110 (11.84)	257 (9.65)	

^a^ Body mass index is calculated as weight in kilograms divided by height in meters squared.

At baseline, the participants’ BAs were positively associated with their chronological ages in both cohorts. In addition, the 2 BA measures showed a positive association (eFigure 1 in Supplement 1).

### Association Between High-Altitude Exposure and Biologically Accelerated Aging

After adjusting for covariates such as sociodemographic factors, health behaviors, and existing chronic diseases, the associations between high-altitude exposure and biologically accelerated aging remained statistically significant (Table 2). In the fully adjusted model, compared with those living at low altitudes, participants living at high altitudes presented increases in KDM-BA accelerated aging of 0.85 years (95% CI, 0.46-1.23 years; *P* < .001) in the WCNPCS cohort and 0.71 years (95% CI, 0.30-1.13 years; *P* < .001) in the WCHAT cohort. These results agree with those of PhenoAge, showing even larger effect sizes (WCNPCS, β, 2.08 years; 95% CI, 1.77-2.39 years; *P* < .001; WCHAT, β, 2.23 years; 95% CI, 1.91-2.54 years; *P* < .001). Thus, individuals residing at high altitudes may experience accelerated BA.

**Table 2.** Associations of High-Altitude Exposure With Accelerated Aging Based on Multiple Linear Regression Models

Algorithm	West China Natural Population Cohort				West China Health and Aging Trend			
	Crude model^a^		Adjusted model^b^		Crude model^a^		Adjusted model^b^	
	β (95% CI), y	*P* value	β (95% CI), y	*P* value	β (95% CI), y	*P* value	β (95% CI), y	*P* value
Klemera–Doubal Biological Age acceleration	1.13 (0.75-1.51)	<.001	0.85 (0.46-1.23)	<.001	0.93 (0.49-1.37)	<.001	0.71 (0.30-1.13)	<.001
PhenoAge acceleration	1.53 (1.21-1.84)	<.001	2.08 (1.77-2.39)	<.001	2.09 (1.74-2.45)	<.001	2.23 (1.91-2.54)	<.001

^a^ No covariates were adjusted in the crude model.

^b^ The adjusted model was adjusted for age, gender, marriage, education, dataset, body mass index, smoking, drinking, hypertension, diabetes, chronic obstructive pulmonary disease, and physical activity.

### Subgroup Analyses

Table 3 presents the results of the subgroup analyses and interaction tests according to age, sex, BMI, smoking status, alcohol intake status, and physical activity. The associations between high-altitude exposure and BA measurements were mostly consistent across the subgroups, aligning with the study’s main findings. In the WCNPCS cohort, the subgroup analysis results revealed larger associations among smokers (PhenoAge, β, 2.74 years; 95% CI, 1.96-3.52 years; *P* < .001; *P* for interaction = .02). Although no significant interaction was detected in the WCHAT cohort (*P* for interaction >.05), smokers presented more pronounced signs of accelerated BA.

**Table 3.** Subgroup Analyses of High-Altitude Exposure With Accelerated Aging Based on Multiple Linear Regression Models

Characteristic	West China Natural Population Cohort					West China Health and Aging Trend				
	No.	KDM-BA acceleration, β (95% CI), y	*P *value	PhenoAge acceleration, β (95% CI), y	*P *value	No.	KDM-BA acceleration, β (95% CI), y	*P* value	PhenoAge acceleration, β (95% CI), y	*P* value
Age, y										
<60	6482	0.50 (0.04 to 0.96)	.03	2.51 (2.14 to 2.89)	<.001	1423	0.43 (−0.23 to 1.10)	.20	2.24 (1.72 to 2.77)	<.001
60-70	2318	0.71 (−0.08 to 1.50)	.08	1.41 (0.80 to 2.02)	<.001	1433	1.14 (0.51 to 1.78)	<.001	2.42 (1.94 to 2.90)	<.001
≥70	1046	2.67 (1.24 to 4.10)	<.001	2.03 (0.86 to 3.20)	<.001	737	0.73 (−0.23 to 1.69)	.14	1.65 (0.92 to 2.37)	<.001
*P* value for interaction^a^	NA	NA	.05	NA	<.001	NA	NA	.38	NA	.44
Sex										
Male	3116	0.46 (−0.23 to 1.15)	.19	2.17 (1.62 to 2.73)	<.001	1340	0.61 (−0.08 to 1.29)	.08	2.68 (2.10 to 3.26)	<.001
Female	6730	1.18 (0.67 to 1.69)	<.001	1.90 (1.49 to 2.31)	<.001	2253	0.71 (0.20 to 1.23)	.007	1.95 (1.58 to 2.32)	<.001
*P* value for interaction^a^	NA	NA	.12	NA	.13	NA	NA	.55	NA	.05
Body mass index^b^										
<24	4522	0.33 (−0.26 to 0.92)	.28	2.16 (1.68 to 2.63)	<.001	1396	0.74 (0.08 to 1.41)	.03	2.58 (2.07 to 3.10)	<.001
24-28	3985	1.40 (0.79 to 2.02)	<.001	2.03 (1.54 to 2.52)	<.001	1429	0.45 (−0.19 to 1.08)	.17	1.99 (1.52 to 2.47)	<.001
≥28	1339	.75 (−0.16 to 1.67)	.11	1.95 (1.20 to 2.71)	<.001	768	1.16 (0.18 to 2.15)	.02	1.94 (1.17 to 2.71)	<.001
*P* value for interaction^a^	NA	NA	.10	NA	.69	NA	NA	.46	NA	.28
Smoking										
No	7963	0.91 (0.47 to 1.35)	<.001	1.88 (1.53 to 2.23)	<.001	2947	0.62 (0.16 to 1.08)	.009	2.09 (1.75 to 2.44)	<.001
Yes	1883	0.91 (−0.03 to 1.85)	.06	2.74 (1.96 to 3.52)	<.001	646	0.93 (0.02 to 1.85)	.05	2.65 (1.85 to 3.45)	<.001
*P* value for interaction^a^	NA	NA	.83	NA	.02	NA	NA	.72	NA	.15
Drinking										
No	6937	1.26 (0.60 to 1.91)	<.001	2.00 (1.48 to 2.53)	<.001	2698	0.68 (0.19 to 1.16)	.006	2.08 (1.71 to 2.44)	<.001
Yes	2909	0.81 (0.26 to 1.36)	.004	2.00 (1.55 to 2.45)	<.001	895	0.63 (−0.16 to 1.43)	.12	2.62 (1.97 to 3.27)	<.001
*P* value for interaction^a^	NA	NA	.16	NA	.80	NA	NA	.82	NA	.19
Physical activity										
<1 Time/wk	1108	0.73 (−1.39 to 2.85)	.50	3.04 (1.30 to 4.79)	<.001	3226	0.77 (0.33 to 1.22)	<.001	2.22 (1.88 to 2.56)	<.001
≥1 Time/wk	8738	0.85 (0.45 to 1.25)	<.001	2.11 (1.79 to 2.43)	<.001	367	0.28 (−0.76 to 1.32)	.60	2.55 (1.71 to 3.38)	<.001
*P* value for interaction^a^	NA	NA	.85	NA	.22	NA	NA	.48	NA	.75

Abbreviations: KDM-BA, Klemera–Doubal Biological Age; NA, not applicable.

^a^ *P* for interaction was calculated using the likelihood ratio test comparing models with and without the interaction term.

^b^ Body mass index is calculated as weight in kilograms divided by height in meters squared.

### Associations Between High-Altitude Exposure and Multidimensional Aging-Related Metrics

**Figure 2.**
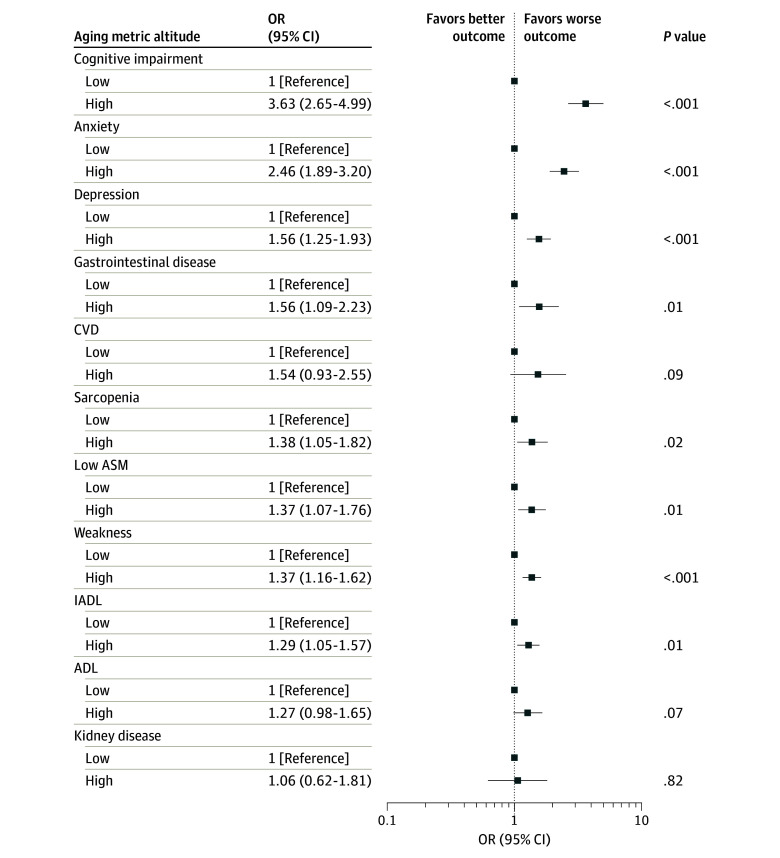
Associations of High-Altitude Exposure With Multidimensional Aging-Related Metrics Based on Multiple Linear Regression Models ADL indicates activity of daily living; ASM, appendicular skeletal muscle mass; CVD, cardiovascular disease; IADL, instrumental activity of daily living; OR, odds ratio.

Figure 2 illustrates the comprehensive analysis results of the associations between high-altitude exposure and various aging-related metrics. Compared with individuals living at low altitudes, those living at high altitudes were more likely to experience cognitive impairment (OR, 3.63; 95% CI, 2.65-4.99; *P* < .001), depression (OR, 1.56; 95% CI, 1.25-1.93; *P* < .001), anxiety (OR, 2.46; 95% CI, 1.89-3.20; *P* < .001), gastrointestinal disease (OR, 1.56; 95% CI, 1.09-2.23; *P* = .01), weakness (OR, 1.37; 95% CI, 1.16-1.62; *P* < .001), sarcopenia (OR, 1.38; 95% CI, 1.05-1.82; *P* = .02), and low ASM (OR, 1.37; 95% CI, 1.07-1.76; *P* = .01). These findings align with the study’s primary results.

### Sensitivity Analyses

A series of sensitivity analyses did not change our primary findings, including regarding AA as a categorical variable with AA equal 0 and AA equal P90 (eTable 1 in Supplement 1), performing propensity score matching (eTable 2 and eTable 3 in Supplement 1), using residual values from regression to gauge BA acceleration (eTable 4 and eTable 5 in Supplement 1), conducting inverse probability weighting (eTable 6 and eTable 7 in Supplement 1), and additionally adjusted for ethnicity, occupation, nutritional status, and medical insurance (eTable 8 and eTable 9 in Supplement 1). In addition, a positive association was observed both between PhenoAge and modified PhenoAge (eFigure 2 and eFigure 3 in Supplement 1), and both metrics demonstrated consistent associations with mortality risk (eTable 10 in Supplement 1). Furthermore, the analyses indicated that high-altitude exposure did not have significant nonlinear associations with accelerated aging (eFigure 4 and eTable 11 in Supplement 1).

## Discussion

This cross-sectional study investigated the associations of high-altitude exposure with accelerated aging and aging-related changes, using data from 2 longitudinal cohorts in Western China. The results showed that people living in high-altitude areas were more likely to experience accelerated aging than their counterparts living at low altitudes. To the best of our knowledge, this is the first large-scale study to investigate the association between high-altitude exposure and biologically accelerated aging. According to the subgroup analyses, this association was particularly high among smokers. Moreover, we observed associations between high-altitude exposure and various multidimensional aging-related metrics. These findings provide robust evidence that high-altitude exposure contributes to AA, and offer novel insights for strategies to mitigate population aging.

High-altitude environments cause oxygen deficiency and atmospheric pressure decrease, leading to hypobaric hypoxia. This condition can produce excessive oxygen radicals, damage cells, and potentially cause cell death^28^; it has also been associated with premature aging.^29^ Prolonged hypoxia induces adaptive changes in high-altitude regions, which are regulated by the hypoxia-inducible factor family at the cellular level.^30^ One of these adaptive changes is hypoxia-inducible factor–1α dysregulation, which is associated with various pathological processes, including aging.^31^ Additionally, Kim et al^32^ reported that people with hypoxia exhibited shortened telomere length and accelerated cellular aging in circulating leukocytes. These findings support our finding that high-altitude exposure is positively associated with accelerated aging, although the underlying mechanisms remain unclear.

Cigarette smoke comprises a complex mixture of chemical constituents, including nicotine, polycyclic aromatic hydrocarbons, heavy metals, carbon monoxide, and other toxic compounds, which induce substantial oxidative stress in cells through the generation of free radicals.^33,34^ Persistent oxidative stress induces functional impairment of autophagy pathways, leading to disrupted cellular signaling and accumulation of damaged proteins, thereby triggering accelerated aging.^35,36^ Moreover, telomeres, which serve as potential biomarkers of aging, undergo progressive shortening following chronic cigarette smoke exposure.^37^ Accordingly, smoking and chronic hypoxia may synergistically act as potent environmental risk factors for accelerated aging and aging-related changes.

Several aging-related changes, such as telomere attrition, mitochondrial dysfunction, and metabolite accumulation, reportedly can trigger cognitive impairment, depression, and anxiety.^7,38^ Weakness is also a common clinical syndrome among the geriatric population, and its prevalence is associated with advancing age.^39^ Consistent with previous research, our study revealed positive associations between high-altitude exposure and multidimensional aging-related metrics, including cognitive impairment, depression, and anxiety.^30,40^ We also found that high-altitude exposure was positively associated with gastrointestinal disease, sarcopenia, and low ASM. Hence, our findings further suggest that high-altitude exposure can accelerate aging.

### Limitations

This study has several limitations that must be noted. First, despite being the first large-scale study examining the association between high-altitude exposure and aging, it cannot elucidate causality because of its observational design. Although we adjusted for numerous variables, the possibility of undisclosed variables still exists. Conducting longitudinal studies with repeated BA assessments could offer further insights. Additionally, the participants were drawn from diverse altitudes in Western China, which boasts a sizable local population. Thus, these results may not be fully generalized to other regions within China or globally. Furthermore, although this research used 2 classic aging measures, it did not incorporate additional or potentially more accurate methods of aging assessment, thereby possibly introducing some bias. Nevertheless, we used multidimensional aging-related metrics to further validate the robustness of our findings.

## Conclusions

In this cross-sectional study of long-term high-altitude exposure with accelerated aging and multidimensional aging-related changes, we presented evidence supporting that extended periods at high altitudes may hasten BA and contribute to the onset of age-related illnesses. Given the global trend of population aging, public health interventions, including maintaining healthy lifestyle, prioritizing antioxidant-enriched dietary patterns, and so on, tailored to individuals residing in high-altitude regions must be implemented to help alleviate the disease burden within these communities.

## Notes

Supplement 1. **eAppendix**. Details of the calculations and formulas for KDM-BA and PhenoAge **eTable 1.** Sensitivity analysis of the main results based on multiple logistic regression models **eTable 2.** Baseline characteristics of the study participants after propensity score matching in WCNPCS **eTable 3.** Associations of high-altitude exposure with accelerated aging based on multiple linear regression models after propensity score matching in WCNPCS **eTable 4.** Ages and aging accelerations of the study participants **eTable 5.** Associations of high-altitude exposure with accelerated aging based on multiple linear regression models **eTable 6.** Baseline characteristics of the study participants in total **eTable 7.** Associations of high-altitude exposure with accelerated aging based on multiple linear regression models after inverse probability weighting **eTable 8.** Supplementary baseline characteristics of the study participants in WCHAT **eTable 9.** Associations of high-altitude exposure with accelerated aging based on multiple linear regression models after adding covariates **eTable 10.** Associations of biological aging measures with mortality **eTable 11.** Associations of different altitudes exposure with accelerated aging based on multiple linear regression models in WCHAT **eFigure 1.** Abstract figure **eFigure 2.** Correlation of Phenoage advancement and Modified Phenoage advancement **eFigure 3.** Correlation of chronological age and two Phenotypic Ages **eFigure 4.** Associations between altitude and its changes with aging acceleration

Supplement 2.
Data Sharing Statement
