# Supplementary material for: Long-Term High-Altitude Exposure, Accelerated Aging, and Multidimensional Aging-Related Changes
Source: JAMA Netw Open. 2025 May 13;8(5):e259960. doi: 10.1001/jamanetworkopen.2025.9960 (PMC12076175; doi:10.1001/jamanetworkopen.2025.9960)
Supplement: Supplement 1. — eAppendix. Details of the calculations and formulas for KDM-BA and PhenoAge eTable 1. Sensitivity analysis of the main results based on multiple logistic regression models eTable 2. Baseline characteristics of the study participants after propensity score matching in WCNPCS eTable 3. Associations of high-altitude exposure with accelerated aging based on multiple linear regression models after propensity score matching in WCNPCS eTable 4. Ages and aging accelerations of the study participants eTable 5. Associations of high-altitude exposure with accelerated aging based on multiple linear regression models eTable 6. Baseline characteristics of the study participants in total eTable 7. Associations of high-altitude exposure with accelerated aging based on multiple linear regression models after inverse probability weighting eTable 8. Supplementary baseline characteristics of the study participants in WCHAT eTable 9. Associations of high-altitude exposure with accelerated aging based on multiple linear regression models after adding covariates eTable 10. Associations of biological aging measures with mortality eTable 11. Associations of different altitudes exposure with accelerated aging based on multiple linear regression models in WCHAT eFigure 1. Abstract figure eFigure 2. Correlation of Phenoage advancement and Modified Phenoage advancement eFigure 3. Correlation of chronological age and two Phenotypic Ages eFigure 4. Associations between altitude and its changes with aging acceleration [file jamanetwopen-e259960-s001.pdf]

Supplemental Online Content

Wu Y, Jin Y, Deng L, et al. Long-term high-altitude exposure, accelerated aging, and multidimensional aging-related changes. *JAMA Netw Open*. 2025;8(5);e259960. doi:10.1001/jamanetworkopen.2025.9960

eAppendix. Details of the calculations and formulas for KDM-BA and PhenoAge
eTable 1. Sensitivity analysis of the main results based on multiple logistic regression models
eTable 2. Baseline characteristics of the study participants after propensity score matching in WCNPCS
eTable 3. Associations of high-altitude exposure with accelerated aging based on multiple linear regression models after propensity score matching in WCNPCS
eTable 4. Ages and aging accelerations of the study participants
eTable 5. Associations of high-altitude exposure with accelerated aging based on multiple linear regression models
eTable 6. Baseline characteristics of the study participants in total
eTable 7. Associations of high-altitude exposure with accelerated aging based on multiple linear regression models after inverse probability weighting
eTable 8. Supplementary baseline characteristics of the study participants in WCHAT
eTable 9. Associations of high-altitude exposure with accelerated aging based on multiple linear regression models after adding covariates
eTable 10. Associations of biological aging measures with mortality
eTable 11. Associations of different altitudes exposure with accelerated aging based on multiple linear regression models in WCHAT
eFigure 1. Abstract figure
eFigure 2. Correlation of Phenoage advancement and Modified Phenoage advancement
eFigure 3. Correlation of chronological age and two Phenotypic Ages
eFigure 4. Associations between altitude and its changes with aging acceleration

This supplemental material has been provided by the authors to give readers additional information about their work.

© 2025 Wu Y et al. *JAMA Network Open*.

eAppendix. Details of the calculations and formulas for KDM-BA and PhenoAge.

1. KDM-BA

An individual's KDM-BA prediction represents the chronological age at which their physiology is considered approximately normal. The KDM-BA is calculated based on a series of regressions of individual biomarkers against chronological age in a reference population. The equation incorporates data from n regression lines, where chronological age is regressed on n biomarkers. The formula is:

$$\mathrm{KDM-BA}_{EC} = \frac{\sum_{i=1}^{n}(x_i - q_i)\frac{k_i}{s_i^2} + \frac{CA}{S_{BA}^2}}{\sum_{i=1}^{n}(\frac{k_i}{s_i})^2 + \frac{1}{s_{BA}^2}}$$

where x is the value of biomarker i measured for an individual. For each biomarker i, the parameters k, q, and s are estimated from a regression of chronological age on the biomarker in the reference sample. k, q, and s are the regression intercept, slope, and root mean squared error, respectively. sBA is a scaling factor equal to the square root of the variance in chronological age explained by the biomarker set in the reference sample. CA is chronological age. The reference sample in the BioAge package consists of nonpregnant NHANES III participants aged 30 to 75 years. Algorithm parameters are estimated separately for men and women. Our study utilized nine biomarkers, including forced expiratory volume in one second (FEV1), systolic blood pressure, albumin, alkaline phosphatase, blood urea nitrogen, creatinine, C-reactive protein, glycated hemoglobin, and total cholesterol.

2. PhenoAge

The PhenoAge algorithm is based on multivariate analysis of mortality risk factors. The original PhenoAge algorithm was developed using elastic-net Gompertz regression of mortality on 42 biomarkers in NHANES III. This analysis identified nine key biomarkers: albumin, alkaline phosphatase, creatinine, C-reactive protein, glucose, mean cell volume, red cell distribution width, white blood cell count, lymphocyte proportion, and chronological age. The formula is:

$$PhenoAge = 141.50225 \frac{ln[-0.00553 \times ln(1 - mortality\ risk)}{0.090165}$$

$$mortality\ risk\ =\ 1 - e^{-e^{xb}[exp(120xy)-1]/\gamma}$$

$$\gamma = 0.0076927$$

$$\begin{aligned} xb = -18.0171 - 0.0239 \times albumin + 0.0067 \times creatinine \\ + 0.1022 \times glucose + 0.1667 \times In(C - reactive\ protein) \\ - 0.0106 \times lymphocyte\ percentage \\ + \ 0.0249 \times mean\ corpuscular\ volume \\ + 0.2384 \times red\ cell\ distribution\ width \\ + 0.0019 \times alkaline\ phosphatase \\ + 0.0601 \times white\ blood\ cell\ count \\ + 0.0782 \times chronological\ age \end{aligned}$$

© 2025 Wu Y et al. *JAMA Network Open*.

eTable 1. Sensitivity analysis of the main results based on multiple logistic regression models.

	WCNPCS				WCHAT			
	Crude Model[a]		Adjusted Model[b]		Crude Model[a]		Adjusted Model[b]	
	OR (95% CI)	P-value	OR (95% CI)	P-value	OR (95% CI)	P-value	OR (95% CI)	P-value
**AA = 0[c]**								
KDM-acceleration	1.43 (1.24, 1.66)	<.001	1.33 (1.12, 1.57)	.001	1.28 (1.08, 1.52)	.005	1.22 (1.01, 1.46)	0.04
PhenoAge-acceleration	2.24 (1.79, 2.82)	<.001	2.74 (2.10, 3.56)	<.001	1.97 (1.69, 2.29)	<.001	2.41 (2.02, 2.86)	<.001
**AA = P90[d]**								
KDM-acceleration	1.90 (1.57, 2.29)	<.001	1.71 (1.36, 2.14)	<.001	1.37 (1.08, 1.75)	0.01	1.37 (1.06, 1.78)	0.02
PhenoAge-acceleration	2.18 (1.62, 2.93)	<.001	2.57 (1.82, 3.61)	<.001	2.09 (1.76, 2.47)	<.001	2.69 (2.21, 3.28)	<.001

OR: odds ratio; AA: aging acceleration

a. Crude Model: no covariates were adjusted
b. Adjusted Model: adjusted for Age, Gender, Marriage, Education, Dataset, BMI, Smoking, Drinking, Hypertension, Diabetes, COPD, Sport
c. AA = 0: the aging acceleration values are further divided into two categories: aging acceleration (AA>0) and non-aging acceleration (AA≤0)
d. AA = P90: the aging acceleration values are further divided into two categories: aging acceleration (AA>P90) and non-aging acceleration (AA≤P90)

© 2025 Wu Y et al. *JAMA Network Open*.

eTable 2. Baseline characteristics of the study participants after propensity score matching in WCNPCS.

Variables	Participants, No. (%)			
	Total	Low altitude	High altitude	P-value
N	1358	679	679	
Age, mean (SD), y	56.96 ± 8.83	56.96 ± 8.83	56.96 ± 8.83	1
KDM-BA acceleration, mean (SD), y	-2.24 ± 5.97	-2.83 ± 5.35	-1.66 ± 6.48	<.001
PhenoAge acceleration, mean (SD), y	-6.87 ± 4.82	-8.19 ± 3.89	-5.55 ± 5.27	<.001
Sex				1
Males	510 (37.56)	255 (37.56)	255 (37.56)	
Females	848 (62.44)	424 (62.44)	424 (62.44)	
Marriage				1
Unmarried	248 (18.26)	124 (18.26)	124 (18.26)	
Married	1110 (81.74)	555 (81.74)	555 (81.74)	
Education				<.001
Primary school	541 (39.84)	245 (36.08)	296 (43.59)	
Junior school	441 (32.47)	201 (29.60)	240 (35.35)	
High school	221 (16.27)	139 (20.47)	82 (12.08)	
College	135 (9.94)	75 (11.05)	60 (8.84)	
Graduate	20 (1.47)	19 (2.80)	1 (0.15)	
BMI				0.04
<18.5	18 (1.33)	11 (1.62)	7 (1.03)	

© 2025 Wu Y et al. *JAMA Network Open*.

>=18.5, <24	518 (38.14)	276 (40.65)	242 (35.64)	
>=24, <28	576 (42.42)	287 (42.27)	289 (42.56)	
>=28	246 (18.11)	105 (15.46)	141 (20.77)	
Smoking				0.38
No	1077 (79.31)	545 (80.27)	532 (78.35)	
Yes	281 (20.69)	134 (19.73)	147 (21.65)	
Drinking				1
No	480 (35.35)	240 (35.35)	240 (35.35)	
Yes	878 (64.65)	439 (64.65)	439 (64.65)	
Hypertension				<.001
No	1083 (79.75)	573 (84.39)	510 (75.11)	
Yes	275 (20.25)	106 (15.61)	169 (24.89)	
Diabetes				0.57
No	1277 (94.04)	641 (94.40)	636 (93.67)	
Yes	81 (5.96)	38 (5.60)	43 (6.33)	
COPD				0.05
No	1354 (99.71)	675 (99.41)	679 (100.00)	
Yes	4 (0.29)	4 (0.59)	0 (0.00)	
Sport				<.001
< 1time/week	147 (10.82)	132 (19.44)	15 (2.21)	
≥1 time/week	1211 (89.18)	547 (80.56)	664 (97.79)	

© 2025 Wu Y et al. *JAMA Network Open*.

eTable 3. Associations of high-altitude exposure with accelerated aging based on multiple linear regression models after propensity score matching in WCNPCS.

	Crude Model[a]		Adjusted Model[b]	
	β (95% CI)	P-value	β (95% CI)	P-value
KDM-acceleration	1.13 (0.75-1.51)	<.001	0.85 (0.46-1.23)	<.001
PhenoAge-acceleration	1.53 (1.21-1.84)	<.001	2.08 (1.77-2.39)	<.001

[a] Crude Model: no covariates were adjusted

[b] Adjusted Model: adjusted for Age, Gender, Marriage, Education, Dataset, BMI, Smoking, Drinking, Hypertension, Diabetes, COPD, Sport

© 2025 Wu Y et al. *JAMA Network Open*.

eTable 4. Ages and aging accelerations of the study participants.

Variables	WCNPCS				WCHAT			
	Total	Low altitude	High altitude	P-value	Total	Low altitude	High altitude	P-value
N	9,846	8996	850		3,593	929	2664	
Age, mean (SD), y	55.73 (11.06)	55.60 (11.22)	57.06 (9.12)	<.001	62.27 (8.40)	63.75 (8.81)	61.76 (8.20)	<.001
KDM-BA acceleration, mean (SD), y[a]	0.41 (5.34)	0.31 (5.23)	1.54 (6.34)	<.001	-0.07 (5.76)	-0.55 (5.80)	0.10 (5.74)	<.001
PhenoAge acceleration, mean (SD), y[a]	0. 33(4.42)	0.20 (4.32)	1.66 (5.10)	<.001	-0.03 (4.79)	-1.67 (4.84)	0.55 (4.64)	<.001

a. Biological aging (BAs) were regressed on their chronological age and residual values from this regression were termed “aging acceleration” (AAs).

© 2025 Wu Y et al. *JAMA Network Open*.

eTable 5. Associations of high-altitude exposure with accelerated aging based on multiple linear regression models.

	WCNPCS				WCHAT			
	Crude Model[a]		Adjusted Model[b]		Crude Model[a]		Adjusted Model[b]	
	β (95% CI)	P-value	β (95% CI)	P-value	β (95% CI)	P-value	β (95% CI)	P-value
KDM-acceleration[c]	1.24 (0.86, 1.61)	<.001	0.85 (0.46, 1.23)	<.001	0.65 (0.22, 1.08)	.003	0.71 (0.30, 1.13)	<.001
PhenoAge-acceleration[c]	1.46 (1.15, 1.77)	<.001	2.08 (1.77, 2.39)	<.001	2.22 (1.87, 2.57)	<.001	2.23 (1.91, 2.54)	<.001

[a] Crude Model: no covariates were adjusted

[b] Adjusted Model: adjusted for Age, Gender, Marriage, Education, Dataset, BMI, Smoking, Drinking, Hypertension, Diabetes, COPD, Sport

[c] Biological aging (BAs) were regressed on their chronological age and residual values from this regression were termed "aging acceleration" (AAs).

© 2025 Wu Y et al. *JAMA Network Open*.

eTable 6. Baseline characteristics of the study participants in total.

Variables	Participants, No. (%)							
	WCNPCS				WCHAT			
	Total	included	excluded	P value	Total	included	excluded	P value
N	33,225	9,846	23,379		7,536	3593	3,943	
Age, mean (SD), y	56.91 ± 11.54	55.6 ± 11.0	57.6 ± 11.7	<.001	56.96 ± 5.45	62.23 ± 8.39	56.80 ± 5.25	<.001
KDM-BA acceleration, mean (SD), y	-2.96 ± 5.51	-2.5 ± 5.3	-3.2 ± 5.6	<.001	-2.48 ± 5.98	-2.52 ± 5.88	-2.48 ± 6.00	.84
PhenoAge acceleration, mean (SD), y	-7.43 ± 4.57	-7.3 ± 4.5	-7.6 ± 4.7	<.001	3.26 ± 2.57	1.05 ± 4.81	3.33 ± 2.43	<.001
Sex				<.001				<.001
Males	34.31	31.6	35.6		82.98	37.41	84.35	
Females	65.69	68.4	64.4		17.02	62.59	15.65	
Marriage				<.001				.56
Unmarried	45.38	39.8	48.5		17.21	15.83	17.4	
Married	54.62	60.2	51.5		82.79	84.17	82.6	
Education				<.001				.22
Primary school	56.03	61	53.5		60.5	64.23	59.96	
Junior school	23.54	18.3	26.2		20.4	22.29	20.13	
High school	10.65	10.4	10.8		13.41	9.59	13.96	
College	7.49	6.6	7.9		5.69	3.86	5.96	
Graduate	2.29	3.7	1.6		0	0.03		
BMI				<.001				<.001

© 2025 Wu Y et al. *JAMA Network Open*.

<18.5	2.35	1.9	2.7		0.87	2.15	0.83	
>=18.5, <24	44.98	44	45.7		83.95	36.63	85.4	
>=24, <28	39.21	40.6	38.2		9.04	39.65	8.1	
>=28	13.47	13.6	13.4		6.15	21.56	5.67	
Smoking				.13				.15
No	80.69	81.2	80.4		78.36	82.13	77.81	
Yes	19.31	18.8	19.6		21.64	17.87	22.19	
Drinking				.90				.64
No	70.99	70.9	71		73.69	74.97	73.51	
Yes	29.01	29.1	29		26.31	25.03	26.49	
Hypertension				<.001				.34
No	86.1	82.1	88.3		73	75.68	72.61	
Yes	13.9	17.9	11.7		27	24.32	27.39	
Diabetes				.45				.61
No	93.89	94	93.8		92.16	93.03	92.04	
Yes	6.11	6	6.2		7.84	6.97	7.96	
COPD				.70				.62
No	99.3	99.3	99.3		98.55	98.92	98.5	
Yes	0.7	0.7	0.7		1.45	1.08	1.5	
Sport				<.001				.10
< 1time/week	16.53	12.2	19.9		92.54	89.83	92.95	

© 2025 Wu Y et al. *JAMA Network Open*.

≥1 time/week	83.47	87.8	80.1		7.46	10.17	7.05	
Altitude				.009				.04
Low altitude	92.7	92.2	93		19.42	24.18	18.16	
High altitude	7.3	7.8	7		80.58	75.82	81.84	

© 2025 Wu Y et al. *JAMA Network Open*.

eTable 7. Associations of high-altitude exposure with accelerated aging based on multiple linear regression models after inverse probability weighting.

	WCNPCS				WCHAT			
	Crude Model[a]		Adjusted Model[b]		Crude Model[a]		Adjusted Model[b]	
	β (95% CI)	P-value	β (95% CI)	P-value	β (95% CI)	P-value	β (95% CI)	P-value
KDM-acceleration	1.16 (0.76, 1.55)	<.001	0.70 (0.30, 1.09)	<.001	0.70 (0.26, 1.14)	.002	0.77 (0.34, 1.19)	<.001
PhenoAge-acceleration	1.55 (1.23, 1.88)	<.001	2.10 (1.78, 2.42)	<.001	2.20 (1.84, 2.55)	<.001	2.21 (1.89, 2.53)	<.001

[a] Crude Model: no covariates were adjusted

[b] Adjusted Model: adjusted for Age, Gender, Marriage, Education, Dataset, BMI, Smoking, Drinking, Hypertension, Diabetes, COPD, Sport

© 2025 Wu Y et al. *JAMA Network Open*.

eTable 8. Supplementary baseline characteristics of the study participants in WCHAT.

Variables	Participants, No. (%)			
	Total	Low altitude	High altitude	P-value
Ethnicity				<.001
Han	1643 (45.73)	898 (96.66)	745 (27.97)	
Yi	816 (22.71)	4 (0.43)	812 (30.48)	
Mongol	881 (24.52)	26 (2.80)	855 (32.09)	
Tibetan	121 (3.37)	0 (0.00)	121 (4.54)	
Others	132 (3.67)	1 (0.11)	131 (4.92)	
Profession				<.001
Farmer	2398 (66.74)	611 (65.77)	1787 (67.08)	
White collar work	277 (7.71)	37 (3.98)	240 (9.01)	
Industrial worker	320 (8.91)	125 (13.46)	195 (7.32)	
Others	598 (16.64)	156 (16.79)	442 (16.59)	
nutritional degree[a]				<.001
well-nourished	2887 (80.37)	810 (87.28)	2077 (77.97)	
at risk of malnutrition	679 (18.90)	115 (12.39)	564 (21.17)	
malnourished	26 (0.72)	3 (0.32)	23 (0.86)	
Medical insurance				<.001
No	704 (20.14)	109 (12.04)	595 (22.97)	

© 2025 Wu Y et al. *JAMA Network Open*.

Yes	2791 (79.86)	796 (87.96)	1995 (77.03)

a. The revised Mini Nutritional Assessment short-form (MNA-SF) has the three classifications that are the same as for the full MNA: 0 – 7 points: malnourished; 8 - 11 points: at risk of malnutrition; or 12 – 14 points: well-nourished.

© 2025 Wu Y et al. *JAMA Network Open*.

eTable 9. Associations of high-altitude exposure with accelerated aging based on multiple linear regression models after adding covariates.

	Adjusted Model I[a]		Adjusted Model II[b]	
	β (95% CI)	P-value	β (95% CI)	P-value
KDM-acceleration	0.71 (0.30, 1.13)	<.001	0.89 (0.37, 1.41)	<.001
PhenoAge-acceleration	2.23 (1.91, 2.54)	<.001	2.70 (2.31, 3.10)	<.001

[a] Crude Model: adjusted for Age, Gender, Marriage, Education, Dataset, BMI, Smoking, Drinking, Hypertension, Diabetes, COPD, Sport

[b] Adjusted Model: adjusted for Age, Gender, Marriage, Education, Dataset, BMI, Smoking, Drinking, Hypertension, Diabetes, COPD, Sport, Ethnicity, Profession, nutritional degree, Medical insurance

© 2025 Wu Y et al. *JAMA Network Open*.

eTable 10. Associations of biological aging measures with mortality.

	Levine Phenotypic Age		Modified-Levine Phenotypic Age	
	N	HR (95% CI)	N	HR (95% CI)
All	27837	1.52 (1.47-1.57)	27912	1.51 (1.46-1.56)
Sex				
males	13421	1.48 (1.42-1.55)	13449	1.48 (1.41-1.54)
females	14416	1.59 (1.50-1.68)	14463	1.57 (1.49-1.66)
Race				
White	13958	1.58 (1.51-1.65)	13979	1.56 (1.50,1.63)
Black	5176	1.42 (1.32-1.53)	5194	1.41 (1.32-1.52)
Other	8703	1.43 (1.32-1.54)	8739	1.43 (1.32-1.54)

BioAge coefficients in the table are hazard ratios estimated from Cox proportional hazard regressions. Levine Phenotypic Age measures were differenced from chronological age for analysis (i.e.values=BA-CA). These differenced values were then standardized to have M=0,SD=1 separately for males and females within the analysis sample so that effect-sizes are denominated in terms of a sex-specific 1 SD unit increase in biological age advancement. Models included covariates for chronological age and sex. The Levine Phenotypic Age was calculated on nine blood chemistry parameters, namely, albumin, creatinine, C-reactive protein, glucose, mean cell volume, alkaline phosphatase, red cell distribution width, white blood cell count, and lymphocyte proportion. The Modified-Levine Phenotypic Age was deprived from eight blood chemistry parameters, including albumin, creatinine, C-reactive protein, glucose, mean cell volume, red cell distribution width, white blood cell count, and lymphocyte proportion.

© 2025 Wu Y et al. *JAMA Network Open*.

eTable 11. Associations of different altitudes exposure with accelerated aging based on multiple linear regression models in WCHAT.

Altitude (m)	WCHAT							
	KDM-acceleration				PhenoAge-acceleration			
	Crude Model[a]		Adjusted Model[b]		Crude Model[a]		Adjusted Model[b]	
	β (95% CI)	P-value	β (95% CI)	P-value	β (95% CI)	P-value	β (95% CI)	P-value
<1000	0		0		0		0	
1000-2000	1.25 (0.74, 1.77)	<.001	1.23 (0.75, 1.71)	<.001	2.41 (2.00, 2.83)	<.001	2.50 (2.14, 2.87)	<.001
≥2000	0.87 (0.39, 1.34)	<.001	0.45 (-0.00, 0.90)	.05	1.78 (1.40, 2.16)	<.001	1.91 (1.57, 2.26)	<.001

[a] Crude Model: no covariates were adjusted

[b] Adjusted Model: adjusted for Age, Gender, Marriage, Education, Dataset, BMI, Smoking, Drinking, Hypertension, Diabetes, COPD, Sport

© 2025 Wu Y et al. *JAMA Network Open*.

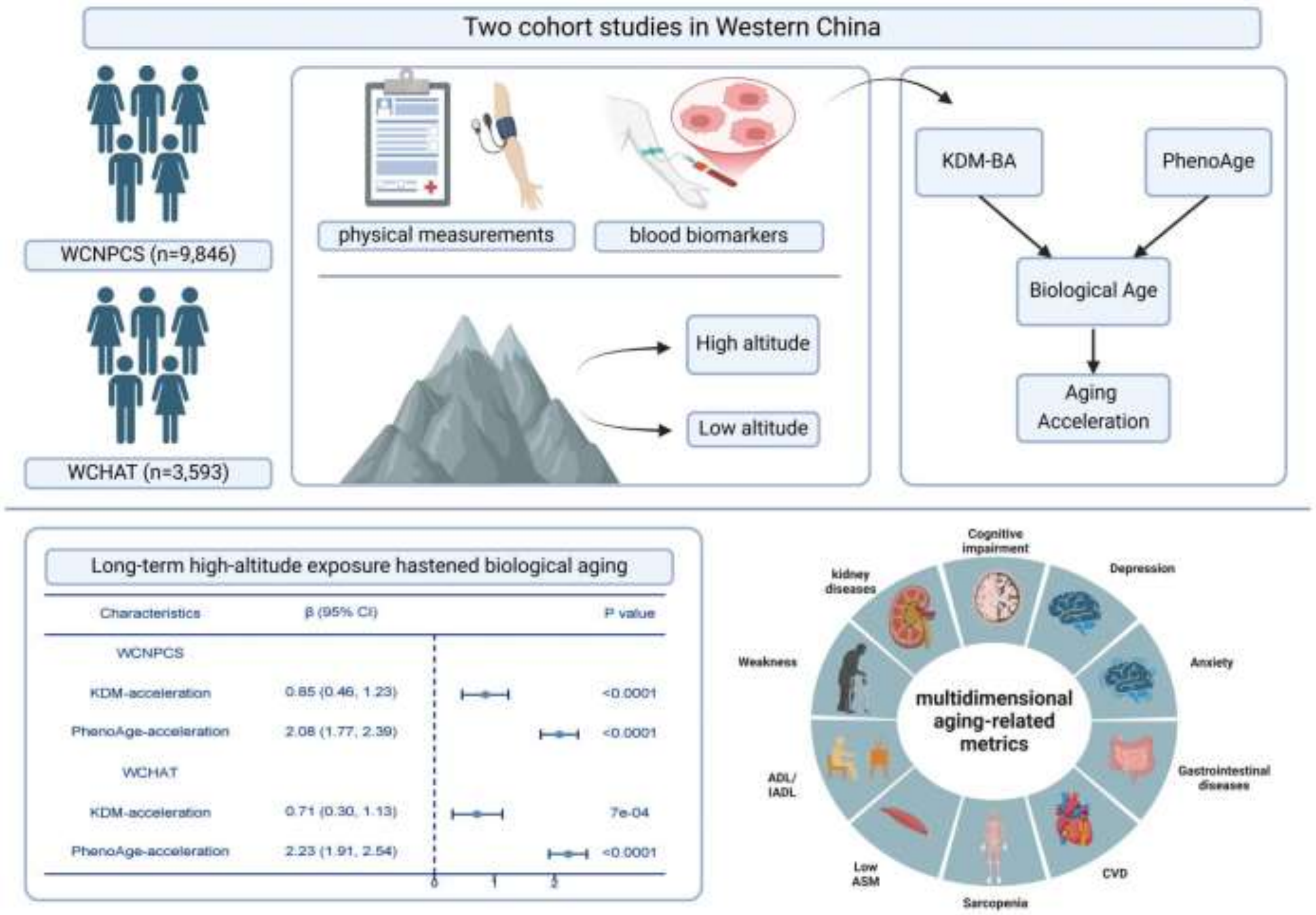

eFigure 1. Abstract figure. This study included 9,846 and 3,593 participants from the WCNPCS and WCHAT cohorts, respectively. We employed the KDM-BA and PhenoAge algorithms to measure the participants' BA based on physical measurement and blood biomarkers. To comprehensively assess the effects of high-altitude exposure on overall aging and related changes.

© 2025 Wu Y et al. *JAMA Network Open*.

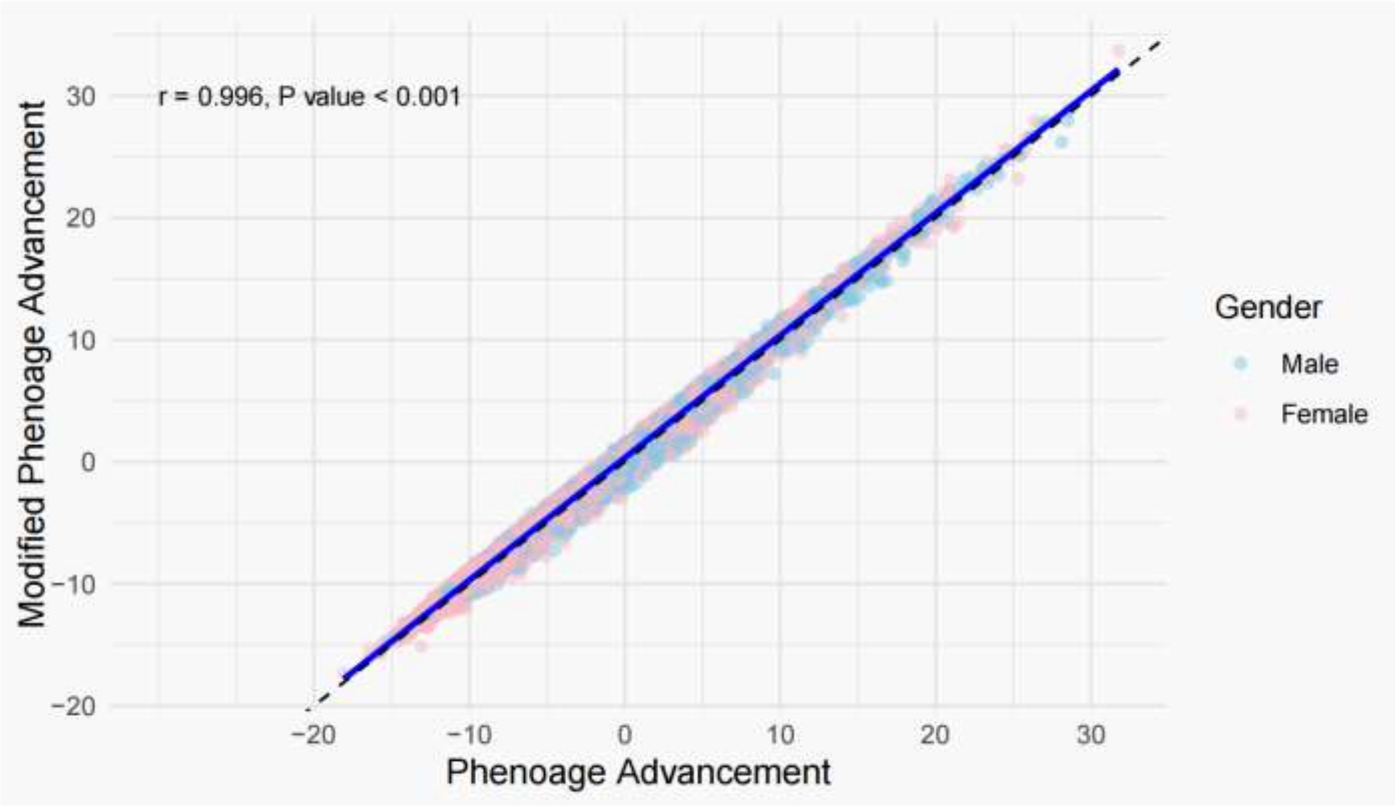

eFigure 2. Correlation of Phenoage advancement and Modified Phenoage advancement.

The Phenoage was calculated on nine blood chemistry parameters, namely, albumin, creatinine, C-reactive protein, glucose, mean cell volume, alkaline phosphatase, red cell distribution width, white blood cell count, and lymphocyte proportion. The Modified Phenoage was deprived from eight blood chemistry parameters, including albumin, creatinine, C-reactive protein, glucose, mean cell volume, red cell distribution width, white blood cell count, and lymphocyte proportion. Phenoage advancement and Modified Phenoage advancement were differenced from chronological age for analysis (i.e.values=BA-CA).

© 2025 Wu Y et al. *JAMA Network Open*.

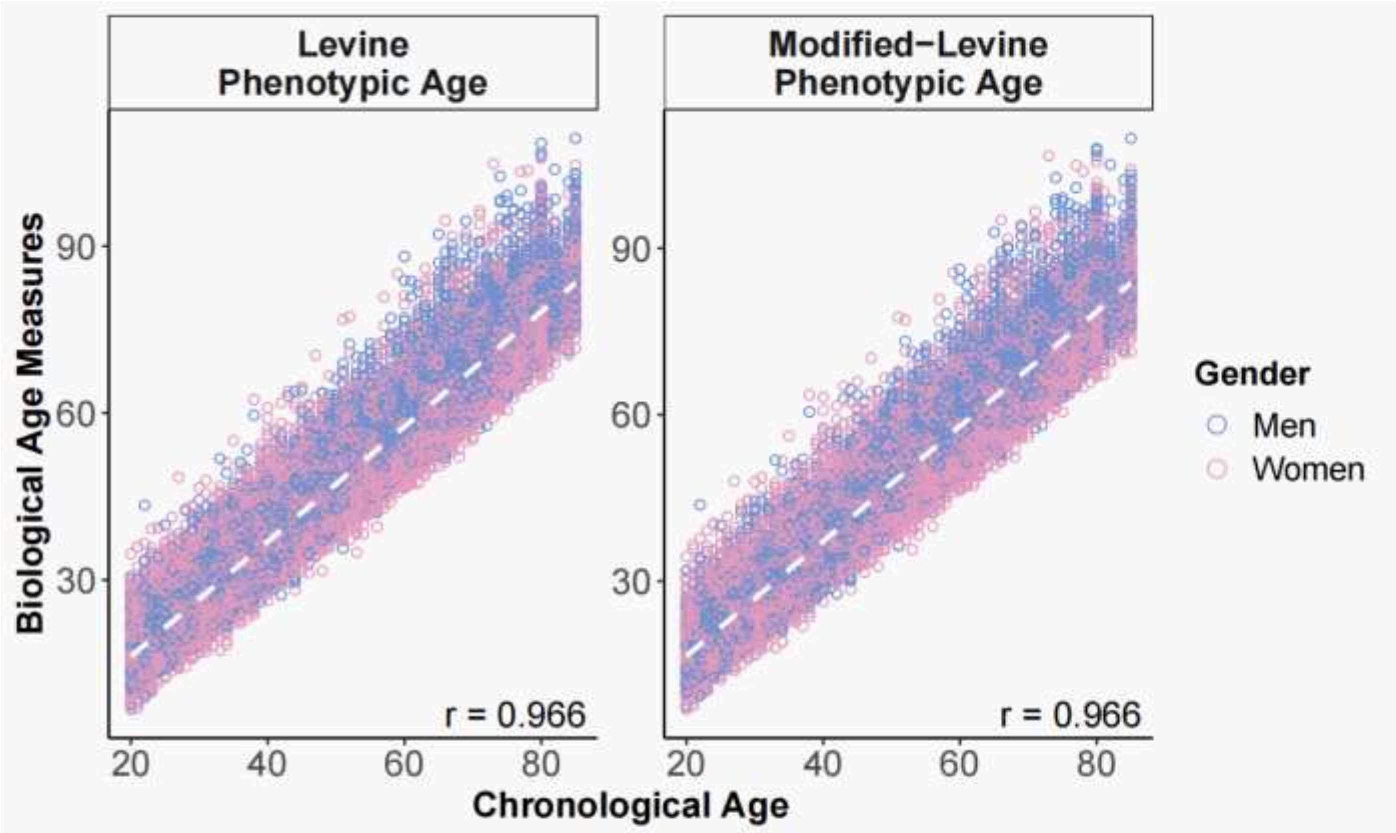

eFigure 3. Correlation of chronological age and two Phenotypic Ages.

The Phenoage was calculated on nine blood chemistry parameters, namely, albumin, creatinine, C-reactive protein, glucose, mean cell volume, alkaline phosphatase, red cell distribution width, white blood cell count, and lymphocyte proportion. The Modified Phenoage was deprived from eight blood chemistry parameters, including albumin, creatinine, C-reactive protein, glucose, mean cell volume, red cell distribution width, white blood cell count, and lymphocyte proportion.

© 2025 Wu Y et al. *JAMA Network Open*.

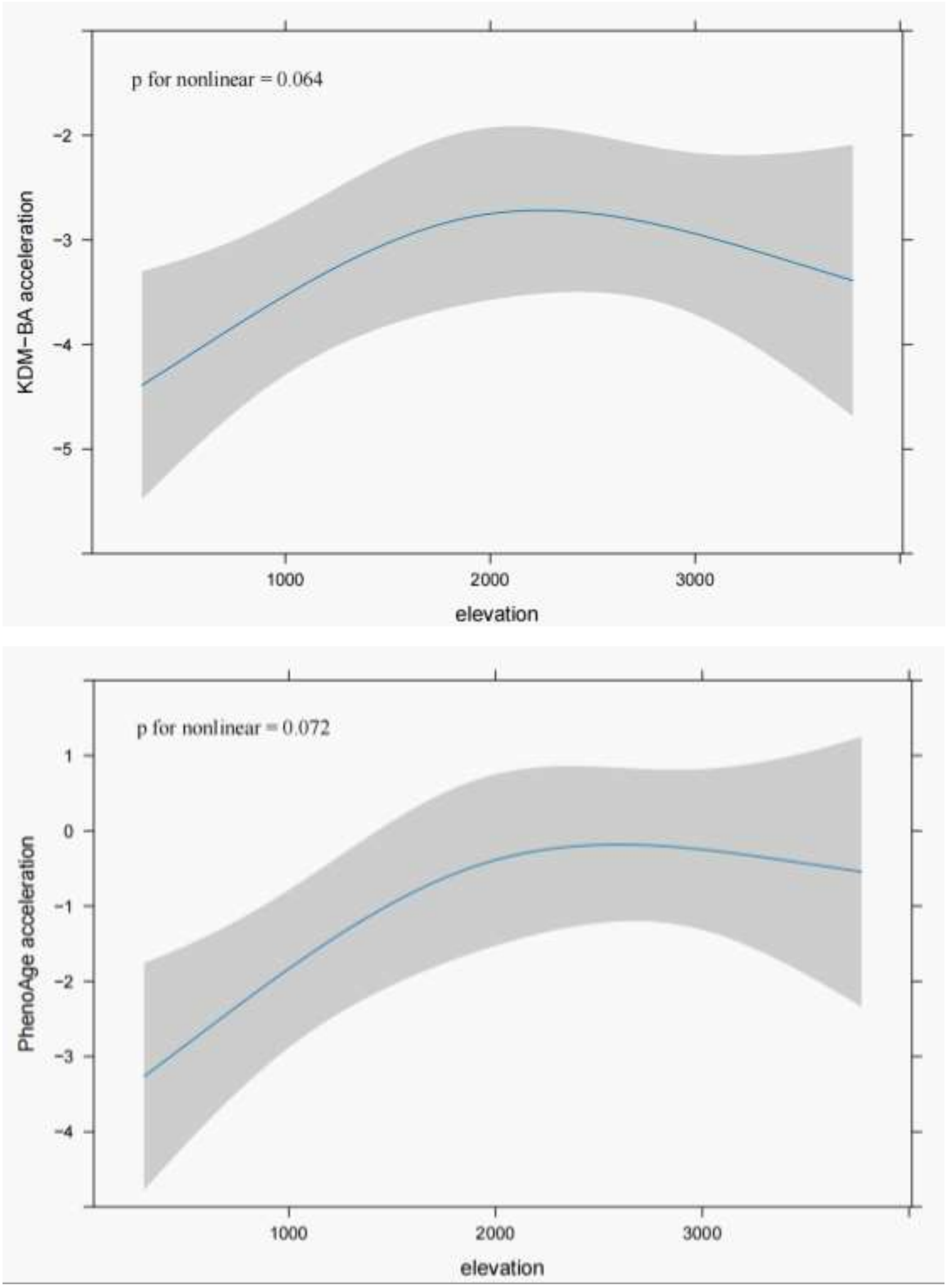

eFigure 4. Associations between altitude and its changes with aging acceleration.

© 2025 Wu Y et al. *JAMA Network Open*.
