# Supplementary material for: Long-Term High-Altitude Exposure, Accelerated Aging, and Multidimensional Aging-Related Changes
Source: JAMA Netw Open. 2025 May 13;8(5):e259960. doi: 10.1001/jamanetworkopen.2025.9960 (PMC12076175; doi:10.1001/jamanetworkopen.2025.9960)
Supplement: Supplement 2. — Data Sharing Statement [file jamanetwopen-e259960-s002.pdf]

Data Sharing Statement

Wu. Long-Term High-Altitude Exposure, Accelerated Aging, and Multidimensional Aging-Related Changes. *JAMA Netw Open*. Published May 13, 2025.
doi:10.1001/jamanetworkopen.2025.9960

Data
Data available: No

Additional Information
Explanation for why data not available: The datasets used and/or analysed during the current study are available from the corresponding author upon reasonable request.
